# Otic Drug Delivery Systems: Current and Future Perspectives

**DOI:** 10.2174/0113816128373749251002102552

**Published:** 2025-10-17

**Authors:** Evren Algın Yapar, İmren Esentürk-Güzel, Merve Nur Özdemir, Lüceyn Abdo

**Affiliations:** 1 Department of Pharmaceutical Technology, Faculty of Pharmacy, Sivas Cumhuriyet University, Sivas, Türkiye;; 2 Department of Pharmaceutical Technology, Hamidiye Faculty of Pharmacy, University of Health Sciences, Istanbul, Türkiye;; 3 Hamidiye Institute of Health Sciences, University of Health Sciences, Istanbul, Türkiye

**Keywords:** Ear disease, otic drug delivery, ML driven drug, nanomedicine, clinical trials, patents

## Abstract

Many people worldwide suffer from various ear diseases, and their treatments are still challenging. The tympanic, round, and oval windows, and the blood-perilymph barrier are the three main physical obstacles to drug delivery. Conventional methods, such as oral administration or injections, often fail to overcome these obstacles. However, local drug delivery systems present a potential solution by reducing side effects and allowing higher drug concentrations to reach the inner ear. Numerous drug delivery techniques and patents have been evaluated in clinical and research settings in recent years. Even though otic drug delivery has evolved, there are still a number of issues, and further study is required to maximize these therapeutic modalities for clinical use. This review summarizes various local drug delivery techniques. Current barriers in otic drug delivery are highlighted, as well as innovative systems for future clinical applications.

## 1 INTRODUCTION

 The ear is a sensitive and structurally well-protected organ, which poses serious difficulties for drug delivery to the inner ear [1]. Traditional drug administration paths, such as oral administration, injections, and topical therapies, have largely demonstrated ineffective treatment due to physical barriers in the ear, including the blood-perilymph barrier, oval window, round window, and the tympanic membrane [2]. Consequently, based on the specific condition and the therapeutic agents employed, localized drug delivery systems can offer substantial benefits compared to systemic administration [3]. Localized administration usually allows higher concentrations of pharmaceuticals to reach the target site, which minimizes side effects. Due to this pharmacokinetic advantage, local administration of drugs has emerged as the preferred clinical strategy for treating inner ear disorders [4, 5].

Now, the core approaches for local inner ear drug delivery include transtympanic, intratympanic, and intracochlear administrations [6]. In recent years, innovative pharmaceutical developments have demonstrated potential in regenerating sensory and nerve cells within the inner ear, which will lead to the development of new therapeutic approaches for hearing loss and other auditory disorders [7, 8]. The nanoparticle drug delivery system deals with molecular property deficiencies, short half-lives, and instability problems in therapies for inner ear diseases. It improves delivery efficiency while reducing drug-related side effects [9, 10].

This review summarizes ear diseases and current therapeutic options, as well as innovative therapeutic approaches focusing on nanomedicine technologies, including patents and clinical trials, with concluding remarks on current challenges and future perspectives.

## 2 METHODS

A comprehensive method was used for the literature search to obtain data on otic drug delivery systems and their current and future perspectives. The search consisted of either the words “local otic drug delivery” or the words “ear barriers” along with “ear diseases” or “otic dosage form” in any field (*i.e*., title, abstract, or keywords). Pubmed, ScienceDirect, ISI Web of Knowledge, and Google Scholar databases were used for research or review articles published in peer-reviewed journals from 2020 to 2025. 1043 titles and abstracts were screened, and 350 full-text articles were evaluated for assessment. 200 articles were excluded according to the eligibility criteria, and the remaining 150 articles were included. For patent searches, the European Patent Office (EPO), the United States Patent and Trademark Office (USPTO), and Google Patents databases were used. The patent search included publication dates between 2009 and 2025, and 152 patents were screened, of which 72 were evaluated and 20 were included. The clinical trials search was conducted on ClinicalTrials.gov (National Library of Medicine, 2024), including studies with a study start date from 2009 to 2025. 364 studies were screened, and 25 of them were evaluated, with 13 of them being included. The PRISMA (Preferred Reporting Items for Systematic Reviews and Meta-Analyses) guidelines have been followed for the preparation of this review article [11], and the PRISMA flowchart is presented in Fig. (**1**). The authors decided together to choose suitable papers based on their titles and abstracts and shared them equally. The risk of bias in the study was independently assessed by 3 review authors using the Cochrane Collaboration’s tool [12]. Sequence generation, allocation concealment, outcome assessment blinding, inadequate outcome data, selective outcome reporting, and other sources of bias were among the categories that were rated. The total risk of bias for each included study was then assessed as either unclear (at least one criterion was assessed to have an unclear risk but no high risk criteria), high (at least one criterion with a high risk of bias), or low (all criteria were assessed to have a low risk).

## 3 STRUCTURAL AND FUNCTIONAL ASPECTS OF THE EAR

 The outer ear, middle ear, and inner ear are the three anatomical divisions of the human ear. The tympanic membrane divides the middle ear from the outer ear, which includes the ear canal and pinna [13]. The middle ear is composed of the ossicular chain, which consists of the malleus, incus, and stapes, and the Eustachian tube, which joins the middle ear to the upper airway and helps regulate pressure [14]. The vestibular system, which regulates balance, and the cochlea, which is in charge of hearing, are both located in the inner ear (Fig. **2**) [15-17].

After entering the pinna and traveling through the ear canal, sound waves cause the tympanic membrane to vibrate. The stapes' motion creates pressure waves that enter the inner ear fluids through the oval window after the ossicles transmit these vibrations [18]. Hair cells in the cochlea detect these vibrations and convert them into electrical impulses, which are conveyed to the brain *via* the cochlear nerve [19, 20].

The Blood-Perilymph Barrier (BPB), the round and oval windows, as well as the tympanic membrane, are significant obstacles to drug delivery to the ear. The tympanic membrane functions as a barrier to the transport of drugs into the middle and inner ears, limiting the flow of most compounds [21]. Round and oval windows serve as the main entry points for medicines into the cochlea and can act as barriers or pathways [22]. The size, electrical charge, concentration, and solubility of the molecule being transported all influence the round window's permeability [23]. Similar to the blood-brain barrier, the BPB only allows for the passage of tiny, lipid-soluble molecules [24]. This barrier, however, inhibits the passage of bigger, water-soluble, or charged molecules from the bloodstream into the cochlea, making systemic drug administration challenging [25]. Due to these factors, the BPB has been recognized as a significant obstacle to the movement of drugs from the systemic circulation to the cochlea [26]. However, inflammation, diuretics, osmotic agents, and noise exposure can all affect the movement of the BPB [27].

To treat inner ear diseases, conventional systemic therapies might not be able to penetrate the BPB. New strategies have been employed to overcome these barriers for efficient drug delivery. The administration of drugs using nanoparticles or hydrogels is one of the most exciting new developments in inner ear therapy. Nanoparticles can be designed to target the inner ear by having functional molecules attach to receptors on inner ear cells. Furthermore, nanoparticles can be made small enough to cross the PBP and protect drugs from the outer and inner ear environment. The application of hydrogels also provides localized administration and prolonged release of therapies inside the inner ear, which may increase their efficacy while reducing systemic toxicity [28]. The use of Artificial Intelligence (AI) to forecast drug delivery to the inner ear is another innovative progress in this field. Artificial Neural Networks (ANN) have shown remarkable accuracy in predicting the distribution and efficacy of drugs, suggesting that they could be a useful tool in pharmaceutical design and development. Additionally, several Machine Learning (ML) methods have been developed to predict the antibiotic dose threshold or target required to achieve a successful therapy, using either the patient's or the antimicrobial agent's properties. Thus, ANN and ML-driven drug delivery techniques to the inner ear minimize side effects and improve the bioavailability of drugs [29].

## 4 EAR DISEASES AND THERAPIES

Many disorders can affect different parts of the ear. Otitis externa, the most common outer-ear condition, is characterized by inflammation of the ear canal, which is frequently caused by bacterial or fungal infections [30, 31]. Symptoms include pain, irritation, discharge, and hearing loss [32]. Middle ear otitis media is a prevalent disease, particularly in children, and can be classified as acute, chronic, or effusion [33-35]. Treatment usually involves pain management and antibiotics, with local antibiotic treatment being considered safer than systemic treatment [36, 37].

 The quality of life is significantly impacted by inner ear conditions, such as autoimmune ear disease, Ménière's disease, tinnitus, and hearing loss [38, 39]. Hearing loss is common among older individuals and can result from various factors, while symptoms of Ménière's disease include tinnitus, vertigo, and fluctuating hearing loss [40, 41]. Treatment for inner ear diseases typically involves corticosteroids, aminoglycosides, and antioxidants [42]. However, systematically administered drugs require high doses to achieve sufficient concentrations in the inner ear, leading to a range of side effects [43]. As a result, local medication administration is becoming more common as a treatment for inner ear disorders [44]. Advances in drug development, along with promising preclinical and clinical studies, offer hope for future treatments of Ménière's disease and tinnitus [45, 46].

## 5 ADMINISTRATION ROUTES FOR OTIC DRUG DELIVERY

Several drug delivery methods have been examined for delivering drugs to the ear. The route of administration is determined by the disease and the drug's physicochemical characteristics. There are two types of drug administration routes into the ear: Systemic and local [47]. This article examines the various drugs that can be delivered locally in the ear.

### 5.1 Local Drug Delivery

 Over the last decades, there has been increasing interest in local drug delivery methods for treating ear diseases, particularly those affecting the inner ear [48]. Localized applications offer several advantages, mainly for medications with a narrow therapeutic window, substantial first-pass metabolism, or significant side effects [49]. Local drug delivery has several significant advantages, including bypassing the Blood-Perilymph Barrier (BPB) [50]. This focused approach also lowers the possibility of systemic adverse effects [51]. Currently, the local delivery methods are: topical, transtympanic, intratympanic, and intracochlear delivery.

 Because the cochlea is especially sensitive to changes in fluid volume and ionic balance, formulations intended for intratympanic or intracochlear distribution must preserve the equilibrium of inner ear fluids [52]. The use of preservatives is limited in local administration to the middle or inner ear [53]. Moreover, preservatives in the middle ear can alter the permeability of the round window and accumulate in the inner ear, potentially causing cochlear cell toxicity [54]. As a result, excipients in locally developed formulations must be safe, biocompatible, and demonstrate minimal immunogenicity [55].

#### 5.1.1 Topical Drug Delivery

Topical drug administration, which involves applying medications directly into the ear canal, has emerged as a significant therapy option for ear disorders. Antibiotics and antifungal drugs are frequently used as topical formulations as foams, gels, or drops [56-58]. The capacity to attain noticeably greater local drug concentrations than systemic administration, which is frequently necessary for successful treatment, is one of the primary benefits of this delivery method [59]. The fast onset of effect, high patient adherence, and the ability to combine several medications in a single formulation are other advantages of topical drug administration. However, the risk of toxicity associated with some drugs, especially at high concentrations, is a notable drawback [60].

Every year, millions of people receive a diagnosis of outer ear infections. Acute otitis externa is a disorder that affects children, adolescents, and sometimes adults. It affects the external auditory canal, which connects the tympanic membrane to the exterior ear. It was reported that the prevalence of the disease is approximately 10%. *Pseudomonas aeruginosa* and *Staphylococcus aureus* are the two microorganisms that cause otitis externa most frequently. The inappropriate use of antibiotics during ear infection therapy causes recurrence and antibiotic resistance [61, 62].

Ear drops are the most common dosage form for otitis externa. However, they might be difficult for proper use since they usually need several treatments a day for seven to ten days, which results in low patient compliance [63, 64]. Therefore, a topical treatment for otitis externa that can be applied once a day has emerged to improve patient compliance and drug bioavailability. Ciprofloxacin otic suspension is the only FDA-approved single-application therapy for otitis externa. The cold-chain dependence of the ciprofloxacin otic solution and the multi-step preparation process, however, raises the possibility of user error. Therefore, Barrett-Catton *et al.* [65] used sodium hyaluronate and hydrolyzed tetraethyl orthosilicate to develop a single-application thixotropic hydrogel that is cold-chain independent for antibiotic administration. The prepared gel's thixotropic qualities allowed it to liquefy during injection, so that the gel would cover the entire ear canal and quickly re-solidify at the infection site. Similarly, Serban *et al.* [66] created two thixogel formulations by mixing large molecular weight biocompatible and biodegradable polymers, such as silk fibroin or glycosaminoglycan, with activated tetraethyl orthosilicate. The thixogel formulations were loaded with ciprofloxacin, gentamicin, or vancomycin once it was established that they are single-application and cold-chain independent. It was found that the efficacy of some antibiotics may be impacted when loaded into thixogels. The MIC values were higher for vancomycin and gentamicin and comparable to those of the ciprofloxacin gel. Antibiotic release rates from thixogels showed that when ciprofloxacin was added to both thixogel formulations, even at doses 100 times lower than those found in commercial products, it exhibited bactericidal effects against both *S. aureus* and *P. aeruginosa*.

Norfloxacin is an antibiotic drug that affects both Gram-negative and Gram-positive bacteria, as well as trimethoprim/ sulfonamide-resistant bacteria [67]. In a study, norfloxacin was loaded into cubosomes, a novel nano-drug carrier with great potential against otitis externa. The lipid phase of the nano-structured cubosomes is glyceryl monooleate, the stabilizer is either Pluronic F108 or Pluronic F127, and the surfactant is Cremophor EL. Due to their bioadhesive properties, norfloxacin-loaded cubosomes were assessed for their capacity to deposit in external ear tissues. The cubosomes that were synthesized measured 216 nm in size, with a zeta potential of -41.2 mV and an excellent polydispersity index of 0.339. *In vivo* experiments show that over the course of 10 hours, a higher concentration of norfloxacin was found in the rabbit ear skin when compared to the drug solution. Additionally, the cubosomes showed no signs of inflammation or irritation on ear skin, which may be safely applied in the treatment of otitis externa [68].

Otomycosis, a fungal infection of the external auditory canal, is a prevalent and recurring disorder that affects millions of people worldwide. Risk factors include the use of topical antibiotics, exposure to humid environments, and injuries to the external auditory canal. The most commonly isolated pathogens are “*Aspergillus niger*” and “*Candida*” species. Treatment failure and recurrence rates for otomycosis are high, often due to patient noncompliance and inadequate use of topical antifungal medications. In one study, 90 patients participated in a randomized controlled trial to evaluate the effectiveness of a foam soaked in povidone-iodine and clotrimazole ointment in treating otomycosis. It was found that using a gel foam soaked in pyodine to treat otomycosis in the external auditory canal is as effective and safe as applying a single clotrimazole ointment topically [69].

Acute otitis media is defined by middle ear inflammation, which is the primary reason for prescribing antibiotics to about 30–40% of children at the age of seven, while 80% of children under three. Recurrent acute otitis media can lead to chronic otitis media, which often requires surgical treatments such as myringotomy and the installation of tympanostomy tubes for drainage and ventilation. Sensorineural dysfunction has also been linked to chronic otitis media. The first topical medication delivery to the middle ear and tympanic membrane without the use of chemical penetration enhancers was researched. A thermoresponsive gel depot was created using poly(lactic-co-glycolic acid) microspheres loaded with the antibiotics ceftriaxone and ciprofloxacin. The results from both *in vitro* and *ex vivo* studies demonstrated that therapeutically significant medication concentrations may be achieved for up to 14 days while still passing through the middle ear and tympanic membrane. Furthermore, research on animals has shown that topical formulations are effective in releasing antibiotics 24 hours after bacterial inoculation [64].

Tinnitus is the ability to perceive sound without actual sound stimulation, which might sound like a ringing sound to many, but it can also sound like a whistling, buzzing, roaring, or screeching sound to others. In fact, it is an auditory system dysfunction, not a disease itself. In research aimed at enhancing a non-invasive oto-topical administration of caroverine to address tinnitus, innovative nano-scale drug delivery systems incorporating phospholipids were created. These systems included nano-elastic vesicles and phosphatidylcholine-derived liquid crystalline nanoparticles. According to the results, phosphatidylcholine-based nanoparticles showed a two-fold cumulative drug value compared to nanoelastic vesicles [70].

Oto-topical drug delivery systems were summarized in Table **1**.

#### 5.1.2 Transtympanic Drug Administration

One important obstacle to the transport of medications to the middle and inner ears is the tympanic membrane. Drugs must diffuse into these inner compartments from the ear canal through the intact tympanic membrane in order for transtympanic medication administration to be effective [71, 72]. Permeation enhancers can help to improve the diffusion process [73]. Recent improvements have demonstrated that transtympanic injection can provide localized and prolonged antibiotic delivery to the middle ear for the treatment of otitis media, particularly when chemical permeation enhancers are used inside a hydrogel matrix [74]. In a study, a combination of drugs was loaded into hydrogels for the treatment of otitis media, which releases both ciprofloxacin and dexamethasone for a period of more than 2-3 weeks, offering important advantages over current treatments in the clinic. Furthermore, the formulations did not exhibit any toxicity in *in vivo* guinea pigs, demonstrating their safety for use in the treatment of otitis media [75]. One of the most frequent infections responsible for otitis media is *Streptococcus pneumoniae* (*S. pneumoniae*). Following a 7-10 day treatment regimen of oral antibiotics, *S. pneumoniae* can be effectively eradicated in the middle ear. However, it becomes difficult for parents to provide drugs to their children orally. Early relapse or treatment failure is linked to noncompliance. Therefore, in another study, a non-invasive transtympanic formulation was developed to overcome this limitation. The thermosensitive *in situ* gelling hydrogel consisted of a temperature-responsive polymer, poloxamer 407-polybutylphosphoester, a combination of 3 chemical enhancers (limonene, sodium dodecyl sulfate, and bupivacaine hydrochloride), and ciprofloxacin hydrochloride as an antibiotic. The direct delivery of ciprofloxacin into the middle ear resulted in significant quantities of the medication on the first day. Throughout the seven-day period of treatment, drug concentrations were higher than the minimum inhibitory concentration for *S. pneumoniae*, and no tissue harm was observed. Thus, using the transtympanic administration route reduced systemic drug exposure [10]. In addition, ciprofloxacin was encapsulated into nano-spanlastic vesicles [76] and nano-transfersomal vesicles [77] for non-invasive transtympanic drug delivery in the treatment of acute otitis media. Recently, in the context of transtympanic vesicular drug carrier systems, for otitis media treatment, niflumic acid was encapsulated into novasomes, which are novel modified nanovesicles that include cholesterol, non-ionic surfactant, and free fatty acids [78]. In another study, levofloxacin was encapsulated into polyethylene glycol-decorated nanoliposomes with similar purposes [79].

Furthermore, using magnetic fields to promote the distribution of magnetic drug nanoparticles has emerged as a viable technique for enhancing drug penetration through the tympanic membrane [80].

 In rat models of otitis media, a recent study revealed that peptides may be actively transported across the intact tympanic membrane, which opens up new possibilities for noninvasive delivery of gene therapy to the middle ear. However, the majority of these technologies are still in the preclinical stage [81-83].

Transtympanic drug delivery systems were summarized in Table **2**.

#### 5.1.3 Intratympanic Drug Administration

In intratympanic drug administration, drugs are administered directly into the middle ear cavity and then diffuse into the cochlea [21, 84]. This diffusion is driven by the concentration difference between the middle ear and the scala tympani, which is located at the opposite side of the RWM [73]. Numerous factors, such as RWM thickness, electrical charge, lipophilicity, concentration gradient, drug structure, and molecular weight, affect the rate of drug diffusion [85].

Studies have reported that the volume of intratympanic injections typically ranges from 0.3 to 0.5 ml [86-88]. While extensive research has been conducted on the RWM as a pathway for inner ear therapies in animal models, there has been limited focus on how interspecies differences affect intratympanic delivery [89]. Factors such as the thickness of the RWM, tissue plugs, bone obstacles, and the existence of fake membranes can all impact the diffusion of substances through the RWM in humans [90]. Therefore, these interspecies variations must be cautiously measured for clinical trial design [91].

Numerous clinical trials observed the use of steroids in the treatment of Ménière's disease and hearing loss. However, this delivery strategy has drawbacks, including anatomical barriers, the potential for drug loss through the Eustachian tube, and uncertain pharmacokinetic profiles of pharmaceuticals administered in this manner. To address these challenges, controlled delivery technologies, such as biodegradable hydrogels and nanoparticles, have been developed to enhance the efficacy of drugs administered intratympanically [92, 93].

Nanoparticles prepared using biodegradable polymers have gained significant attention in recent years. The EMA and FDA have approved poly(lactic-co-glycolic acid) (PLGA) for parenteral delivery, making it a perfect nanomaterial. Therefore, several studies have been carried out using PLGA nanoparticles *via* intratympanic injections. In one of these studies, biodegradable PLGA nanoparticles containing interferon α-2b were incorporated into a chitosan/glycerophosphate-based thermosensitive hydrogel for the treatment of inner ear diseases. The injectable hydrogel exhibited 1.5 to 3-fold swelling and deswelling properties at physiological pH and transitioned into a semi-solid gel at 37°C [94]. In addition, dexamethasone-containing PLGA nanoparticles in thermosensitive gels for inner ear inflammation were prepared [95]. The properties of the nanoparticles' surface have a significant influence on their performance *in vivo* and overall efficacy because of the interactions with biological systems. In another study, coumarin-6 was loaded into PLGA nanoparticles with surface modifications. The developed nanocarrier system enabled drug targeting to the cochlea, which is a potential approach for treating hearing loss [96]. Various other drug carrier systems including solid lipid nanoparticles [97, 98], liposomal gel [99], hollow mesoporous framework capsules [100] and silk fibroin- polyethylene glycol hydrogels [101, 102] were prepared for intratympanic application in the treatment of many ear diseases such as Ménière’s disease, hearing loss, inner ear infections and inflammation, acute noise exposure-induced cochlea damage, and cisplatin-induced ototoxicity.

Intratympanic drug delivery systems are summarized in Table **3**.

#### 5.1.4 Intracochlear Drug Administration

Intracochlear drug delivery effectively bypasses the middle ear by providing a direct route to the inner ear, allowing drugs to reach their target sites with higher efficacy compared to other delivery methods [112]. This approach significantly improves drug bioavailability and can be achieved by a variety of techniques [105].

 Microneedles can be utilized to induce transient microperforations in the Round Window Membrane (RWM), which allows for better diffusion and retention of therapeutic drugs in the cochlear perilymph [113, 114]. The use of microneedles in the RWM enables the formation of temporary microperforations, which demonstrate healing over 24-48 hours without resulting in significant anatomic or physiologic dysfunction and also stabilizing endocochlear pressure variations [115, 116]. Innovative designs for microneedles are being developed to allow precise penetration detection without affecting the integrity of the RWM [117, 118]. In research, polymeric microneedles were developed that allow the guinea pig RWM to be penetrated *in vivo* and perilymph to be aspirated for diagnostic assessment; the RWM is fully restored in 48 to 72 hours. The capacity of microneedles to deliver precise dosages of medications to the cochlea and the ensuing impact on hearing were assessed [115]. Dexamethasone-coated PLGA microneedles were created by Pawley *et al.* [119] as an enhanced drug delivery method for intracochlear biodegradable devices [120].

Osmotic pumps and cochlear implants offer cutting-edge techniques for controlled drug administration of numerous drugs directly into the cochlea, hence increasing therapeutic efficacy [121]. Despite these advantages, intracochlear administration poses various obstacles [122]. One major disadvantage is its intrusive nature, which may cause discomfort and difficulties during the surgery [123]. Furthermore, specific pharmacokinetics of the inner ear may result in toxicity from locally administered drugs. This complexity demands careful attention and monitoring to assure the safety and efficacy of intracochlear drug delivery [124]. In a study, a microfabricated reciprocating micropump was tested for automated inner ear drug delivery and loaded with 6,7-dinitroquinoxaline-2,3-dione, which interferes with synaptic transmission between hair cells and the auditory nerve, resulting in a reduction of the compound action potentials generated in response to auditory stimuli. It was demonstrated that the developed micropump was sufficiently resilient to operate effectively even when hydrodynamic resistance was introduced at the outlet, allowing it to sustain drug delivery under pressures 50 times higher than the standard intracochlear pressure observed in guinea pigs [125]. In another study, biodegradable dexamethasone incorporated PLGA-based implants were prepared by hot-melt extrusion for controlled intracochlear drug release. According to the results, *in vitro* release kinetics showed no significant lag time. Ultimately, the release kinetics aligned well with the expected profile, demonstrating a controlled release for several weeks [126].

Intracochlear drug delivery systems are summarized in Table **4**.

## 6 OTIC DRUG DELIVERY PATENTS

 Patents related to drug formulations used in the treatment of ear diseases are given below. Table **5** highlights recent developments in therapeutic agents and drug formulations.

## 7 CLINICAL TRIALS FOR OTIC FORMULATIONS

Examples of clinical studies on otic formulations are given in Table **6** below. The table includes information on the purpose for which these formulations were used, the stage of the study, and the drugs used in the study.

## 8 CURRENT CHALLENGES AND FUTURE PERSPECTIVES

Recent developments in otic drug administration have made substantial progress in understanding the challenging anatomy of the ear and the molecular pathways of inner ear disorders [150-153]. Diseases like sensorineural Ménière’s disease, hearing loss, and tinnitus pose substantial challenges due to our limited understanding of their pathophysiology. Improved comprehension of inner ear anatomy and disease pathology promises to enhance the efficacy of otic drug treatments [154, 155].

The pharmacokinetics of medicines in the inner ear are still poorly understood, with limited information on round window permeability, local bioavailability, and drug dispersion [156]. Standardized methods for evaluating drug delivery to the inner ear are lacking, unlike established techniques for transdermal formulations [21, 84, 157]. *In vitro* diffusion cell systems may not adequately simulate the middle ear and RWM drug diffusion, which highlights the need for a suitable evaluation method for intratympanic formulations [158].

Additionally, the inner ear's tiny fluid volume impacts traditional analytical sample procedures. LC-MS techniques provide sensitivity, but they are complex and expensive, making it difficult to measure drug concentrations in cochlear fluids [159, 160]. Regardless of these challenges, understanding the pharmacokinetics of inner ear medications is crucial for improving clinical practices and designing successful treatments [156, 161].

Empirical approaches currently guide the selection of drugs and application protocols for inner ear delivery in clinical trials. However, there is a growing need to improve *in vitro–in vivo* correlations for otic drugs, given anatomical and physiological differences between species and variations in RWM permeability and cochlear clearance [162]. Future research should focus on developing *in vitro* testing methods for inner ear drug delivery.

Furthermore, the safety and toxicity profiles of new inner ear drug delivery methods are not well known. The complexity of the inner ear emphasizes the significance of thoroughly examining potential side effects, as problems may result in significant consequences such as hearing loss [163]. Vigilant investigation of ototoxicity hazards is critical for the advancement of these new delivery methods [164, 165].

In terms of studies conducted for local otic drug delivery, it was observed that nanoparticulate systems were more successful in drug delivery than other carrier systems or extended-release polymeric systems, and that they provided advantages in overcoming the difficulties of the anatomical structure of the ear. In addition, it has been stated that dosage designs that increase the passage of active substances from the dosage form to the inner ear (microneedle application, magnetic activation, *etc*.) are applications that increase success. When administering local otic drugs for future studies, loading active substances into nanocarrier systems, adapting drug targeting mechanisms to formulations, and using applicators that will increase the delivery of the formulation to the target tissue can be suggested as approaches to increase success.

The latest research confirms this prediction. While one of the latest articles in the field examines the delivery of ciprofloxacin and paclitaxel from nanofibers, a type of nanocarrier in local ear drug release [166], another one is postauricular injection administration [167] as a new application method for drug delivery to the inner ear to overcome the anatomical barriers of the ear. On the other hand, a new alternative animal model has been reported, demonstrating the feasibility of perilymph sampling in the Mongolian gerbil as a new animal model for evaluating the pharmacokinetics of otoprotective drugs [168].

An important step on the path to the above-mentioned developments becoming a final product is the field of legal regulation. While scientific and technological developments in the field of nanotechnology play an encouraging role in the development and approval of non-biological complex drugs, the speed of developments in nanotechnology creates difficulties in terms of legal regulations [169]. In addition, different approval mechanisms for the same product group among different regulatory authorities around the world make the international acceptance of products difficult [169, 170]. It is stated that the American Food and Drug Agency is working to reduce the obstacles of complex generic drug development, and that in its published definition of "complex generic drug products," it includes otic dosage forms formulated as suspensions, emulsions, or gels in this group. On the legal regulation side, especially local otic drug delivery, presenting bioavailability studies appears to be a challenging step due to the anatomical barriers of the ear [170].

## CONCLUSION

Otic drug delivery applications have made notable progress in recent years. Over the past decade, diverse systemic and local drug delivery systems have been developed.

Systemic drug delivery remains an important strategy in otic treatment due to its minimally invasive nature and convenience for self-administration, despite the potential for side effects. This approach continues to play a significant role in addressing ear-related conditions.

Local drug delivery in the ear offers the advantage of delivering high therapeutic concentrations directly to the site of action while minimizing systemic side effects. To optimize effectiveness, the method of administration should be tailored to the patient's specific condition, taking into account possible side effects and individual needs.

The formulation of otic treatments must align with key principles to guarantee accuracy and effective targeting for managing diverse auditory conditions. Although hurdles remain in the development of otic medications, progress in comprehending ear physiology has paved the way for cutting-edge delivery mechanisms, including hydrogels, nanoparticles, and other minimally invasive techniques. These innovations offer improved therapeutic solutions with reduced adverse effects, significantly enhancing the overall well-being of individuals affected by ear-related disorders.

In conclusion, although progress has been made in otic drug delivery, these issues must be addressed through a better understanding of inner ear biology, improved pharmacokinetic assessments and rigorous safety evaluations, increased studies of newly proposed *in vivo* animal models, the use of nanocarrier systems including tissue targeting in formulations, and the use of microneedle drug delivery systems that increase drug penetration into the inner ear may pave the way for more successful treatments in clinical practice.

## Figures and Tables

**Fig. (1):**
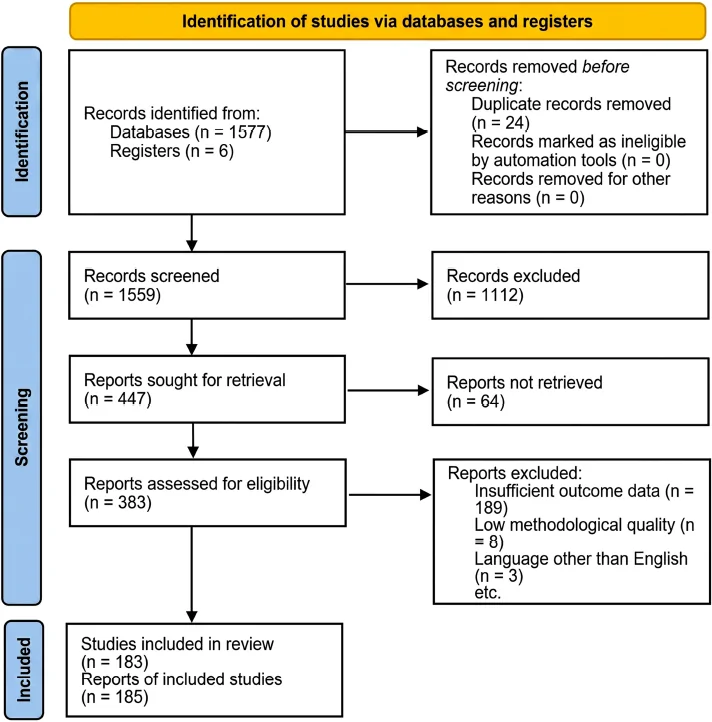
PRISMA flowchart of data acquisition and examination.

**Fig. (2):**
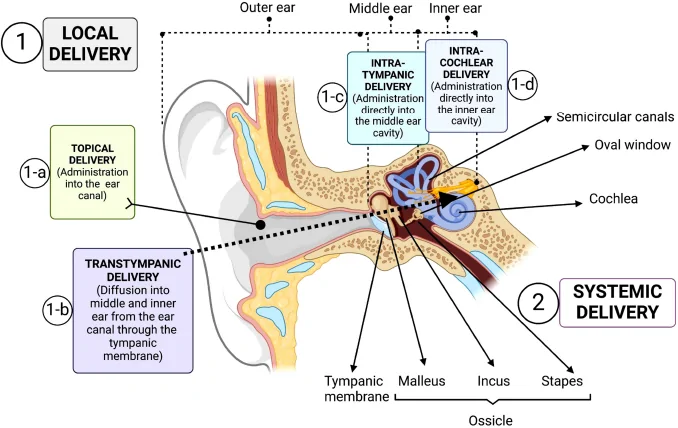
Anatomy of the human ear (created with BioRender.com).

**Table 1:** Ototopical drug delivery systems.

**Disease**	**Drug**	**Drug Delivery System**	**Route of Administration**	**Aim of the Study**	**References**
Otitis externa	Norfloxacin	Nano-cubosome	Topical	The research investigates the use of norfloxacin-loaded nano-cubosomes for the treatment of otitis externa, showing improved efficacy both *in vitro* and *in vivo*. This topical delivery system offers improved drug management for otitis externa.	[61]
Otitis externa	Gentamycin	Thixotropic hydrogel	Topical	This study explores a thixotropic hydrogel for the controlled release of gentamycin to treat otitis externa. The hydrogel is designed for single-application therapy, providing an effective topical treatment option for the condition.	[62]
Acute otitis media	Ciprofloxacin and ceftriaxone	Thermoresponsive gel depot containing antibiotic-loaded microspheres	Topical	This research investigates a controlled release system using ciprofloxacin and ceftriaxone-loaded polymer microspheres within a thermoresponsive gel. The system allows for a single ototopical application with sustained release, enhancing treatment for acute otitis media.	[64]
Otitis externa	Hyaluronidase	Hydrogel	Topical	This study examines a hydrogel based on tetraethyl orthosilicate for treating otitis externa with a single application. The hydrogel aims to improve the manufacturability and effectiveness of topical therapies for ear infections.	[65]
Otitis externa	Ciprofloxacin	Thixogels	Topical	The study evaluates a cold-chain-independent drug delivery system using ciprofloxacin-loaded thixogels for the treatment of otitis externa. The system provides a single-application, stable topical treatment for outer ear infections.	[66]
Otomycosis	Clotrimazole and povidone-iodine	Gelfoam	Topical	This randomized controlled trial compares the effectiveness of gel foam soaked in pyodine and the topical application of clotrimazole for treating otomycosis. Results suggest that both treatments are effective, with pyodine-soaked gelfoam offering an alternative and enhanced approach.	[68]
Tinnitus	Caroverine	Nanoelastic vesicles and phosphatidylcholine-based liquid crystalline	Topical	This study explores caroverine-loaded phosphatidylcholine-based nanocarrier for tinnitus treatment. The delivery system improves drug stability, characterization, and *ex vivo* transport, offering potential for more effective treatment.	[69]
Otitis media	Peptide P60.4Ac	Drops	Topical	This research evaluates the safety and efficacy of ototopical drops containing a synthetic peptide (P60.4Ac) for chronic otitis media. The peptide-based drops provide a promising treatment option for this ear condition.	[70]
Otitis externa	Polymyxin B sulfate, neomycin sulfate, and gramicidin combination	Drops	Topical	This study compares the safety and efficacy of antibiotic ear drops (polymyxin B sulfate, neomycin sulfate, and gramicidin) with glycerol treatment for acute bacterial otitis externa. The antibiotic drops demonstrate superior efficacy for treating the condition.	[71]

**Table 2:** Transtympanic drug delivery systems.

**Disease**	**Drug**	**Drug Delivery System**	**Route of Administration**	**Aim of the Study**	**References**
*Streptococcus pneumoniae* otitis media	Ciprofloxacin	Thermosensitive *in situ* gelling hydrogel	Transtympanic	This study investigates the use of an *in situ* gel for the transtympanic delivery of ciprofloxacin to treat *S. pneumoniae* infection in a chinchilla model. The system enhances localized drug release and effectiveness against bacterial otitis media.	[10]
Otitis media	Ciprofloxacin	Hydrogel	Transtympanic	This study focuses on the transtympanic delivery of ciprofloxacin using a hydrogel formulation. The approach enhances middle ear drug delivery, offering an effective therapeutic option for ear infections.	[73]
Otitis media	Ciprofloxacin/ Dexamethasone	Hydrogel	Transtympanic	This research examines ciprofloxacin and dexamethasone-loaded hydrogel for transtympanic delivery in treating otitis media. The dual-drug hydrogel provides sustained release, improving the therapeutic outcomes for patients with otitis media.	[75]
Acute otitis media	Ciprofloxacin	Nano-spanlastics	Transtympanic	This research uses nano-spanlastic vesicles for improved non-invasive transtympanic delivery in acute otitis media. The system demonstrated effective drug permeation and favorable *in vitro* and *ex vivo* results.	[76]
Acute otitis media	Ciprofloxacin	Nano-transfersomal vesicles	Transtympanic	This study investigates the use of nano-transfersomal ciprofloxacin-loaded vesicles for transtympanic ototopical delivery. The optimized vesicles demonstrated improved drug permeation, *in vitro* performance, and *in vivo* efficacy for acute otitis media treatment.	[77]
Acute otitis media	Niflumic acid	Novasomes	Transtympanic	This research examines the potential of novasomes in enhancing the trans-tympanic delivery of niflumic acid for the treatment of acute otitis media. The novasome-based formulation improves drug transport and effectiveness, offering a promising treatment alternative.	[78]
Acute otitis media	Levofloxacin	Polyethylene glycol decorated nanoliposomes	Transtympanic	This article details the development of polyethylene glycol-decorated nanoliposomes loaded with levofloxacin for transtympanic delivery in acute otitis media. The optimized formulation shows improved permeation and therapeutic efficacy, supported by *in vivo* evaluation.	[79]
Otitis media	Norfloxacin	Reverse gelatination gel	Transtympanic	This study applies Quality by Design (QbD) principles to optimize a reverse gelling formulation of norfloxacin for transtympanic delivery. The research investigates gene interactions to improve otitis media treatment.	[83]

**Table 3:** Intratympanic drug delivery systems.

**Disease**	**Drug**	**Drug Delivery System**	**Route of Administration**	**Aim of the Study**	**References**
Inner ear diseases	Interferon α-2 b	Thermosensitive hydrogel loaded with PLGA nanoparticles	Intratympanic	This study highlights the development of an injectable, biodegradable hydrogel integrated with PLGA nanoparticles for drug delivery into the inner ear. This formulation aims to improve both the efficiency and duration of drug release, offering an innovative approach for addressing inner ear conditions.	[94]
Hearing loss	Coumarin-6	Surface-modified nanoparticles	Intratympanic	This article focuses on optimizing poly(lactic-co-glycolic acid) (PLGA) nanoparticles modified with hydrophilic molecules to improve drug delivery to the cochlear outer hair cells. This method aims to boost the therapeutic efficiency in treating various forms of hearing impairment.	[96]
Acute noise exposure-induced cochlear damage	Clozapine	Solid lipid nanoparticles	Intratympanic	This research investigates the use of Solid Lipid Nanoparticles (SLNs) encapsulating clozapine as a treatment for cochlear damage caused by acute noise exposure. These SLNs enable targeted drug transport to the cochlea, which may help reduce the harmful impact of noise-induced injury.	[97]
Protecting auditory cells from cisplatin-induced ototoxicity	Dexamethasone and hydrocortisone	Solid lipid nanoparticles	Intratympanic	This study examines the use of SLNs containing dexamethasone and hydrocortisone to shield auditory cells from cisplatin-induced toxicity. The nanoparticles enhance targeted delivery and provide sustained release of glucocorticoids, potentially offering protection against hearing loss triggered by ototoxic medications.	[98]
Residual hearing	N-acetyl-L-cysteine	NAC-loaded liposomal gel	Intratympanic	This research explores the intratympanic delivery of N-acetyl-L-cysteine (NAC) in a liposomal gel for preventing cochlear damage following implantation in guinea pigs. The study aims to assess whether this strategy can preserve residual hearing after cochlear implantation.	[99]
Ménière’s disease	Gentamicin	Hollow mesoporous silica at a zeolitic imidazolate framework capsule	Intratympanic	This article explores the development of hollow mesoporous silica-based capsules combined with zeolitic imidazolate frameworks for the delivery of gentamicin in the management of Ménière’s disease. The system is designed to achieve controlled drug release and precise targeting, potentially enhancing therapeutic results for individuals suffering from inner ear disorders.	[100]
Inner ear diseases	Micronized dexamethasone	Silk fibroin- polyethylene glycol hydrogel	Intratympanic	This research investigates the use of a silk-polyethylene glycol (PEG) hydrogel loaded with dexamethasone for the treatment of inner ear diseases. The injectable hydrogel provides sustained release of dexamethasone, offering improved therapeutic outcomes.	[101]
Cisplatin-induced ototoxicity	Dexamethasone	Injectable silk-polyethylene glycol hydrogel	Intratympanic	This research investigates a silk-polyethylene glycol hydrogel loaded with dexamethasone for intratympanic delivery to alleviate cisplatin-induced ototoxicity. The injectable hydrogel provides controlled release, improving the treatment of hearing loss caused by chemotherapy.	[102]
Cisplatin-induced ototoxicity	N-acetylcysteine	Thermosensitive hydrogel	Intratympanic	This study demonstrates the use of poloxamer 407 hydrogels for the intratympanic delivery of N-acetylcysteine in treating cisplatin-induced ototoxicity. The formulation ensures sustained release and enhanced drug efficacy for protecting cochlear cells.	[103]
Inner ear diseases	Dexamethasone and gene therapy	Nanoparticles	Intratympanic	This article evaluates gene delivery to the inner ear using dexamethasone and peptide nanoparticles. The nanoparticles aim to enhance the delivery of therapeutic genes and drugs, offering a novel approach to treating inner ear diseases.	[104]
Inner ear diseases	Oligoarginine	Nanoparticles	Intratympanic	This study explores the use of oligoarginine-conjugated nanoparticles for intratympanic delivery of genes and drugs to the inner ear. The system enhances targeted delivery and cellular uptake, potentially improving treatments for inner ear disorders.	[105]
Inner ear disorders	Dexamethasone	Hydrogel	Intratympanic	This article examines injectable poloxamer hydrogel formulations for the intratympanic delivery of dexamethasone. The hydrogels ensure controlled release and localized treatment for various inner ear disorders, offering enhanced therapeutic potential.	[106]
Hearing loss	Dexamethasone	Nanosuspension	Intratympanic	This study evaluates the intratympanic injection of a dexamethasone nanosuspension in order to treat acute hearing loss. The nanosuspension improves drug solubility and bioavailability, offering a potential solution for acute ear conditions.	[107]
Hearing loss	Dexamethasone	Gel	Intratympanic	This article investigates a gel-based dexamethasone formulation. The study shows that the gel-based system improves drug retention in the inner ear, preserving cochlear function and providing better treatment for hearing loss.	[108]
Hearing loss	Dexamethasone	Phospholipid-based nanoparticle	Intratympanic	This study explores the application of optimized phospholipid-based nanoparticles for delivering dexamethasone to the inner ear. The formulation improves the drug's bioavailability and enhances its therapeutic effectiveness in treating hearing loss.	[109]
Inner ear disorders	Dexamethasone	Solid lipid nanoparticles	Intratympanic	This study investigates the *in vitro* release of dexamethasone from nanoparticles and their pharmacokinetics following intratympanic administration *via* the round window. The results highlight the sustained drug release and optimized pharmacokinetics for treating inner ear disorders.	[110]
Hearing loss	Disulfiram	Liposome and polymersome nanoparticles	Intratympanic	This article explores the application of liposome and polymersome nanoparticles for minimally invasive drug delivery to the cochlea through the round window membrane. The system enhances drug permeability and efficacy in treating hearing loss.	[111]

**Table 4:** Intracochlear drug delivery systems.

**Disease**	**Drug**	**Drug Delivery System**	**Route of Administration**	**Aim of the Study**	**References**
Inner ear disorders	Stains as drug models	Microneedles	Intracochlear	This research demonstrates the use of microneedles for small-volume, intracochlear drug delivery in guinea pigs. The approach ensures precise delivery without causing physiological injury, offering a minimally invasive method for treating inner ear disorders.	[115]
Hearing loss	Dexamethasone	Microneedle	Intracochlear	This article explores dexamethasone-coated PLGA microneedles for intracochlear delivery to treat hearing loss.	[119]
Hearing loss	siRNA and Lipofectamine	Liposome containing microneedle	Intracochlear	This study investigates microneedles loaded with siRNA-encapsulated liposomes for gene therapy targeting the inner ear. The approach targets hearing loss by enabling efficient gene delivery directly to the cochlea.	[120]
Inner ear disorders	6,7-dinitroquino-xaline-2,3-dione	Drug-containing micropump	Intracochlear	This article introduces a microengineered reciprocating micropump system designed for precise and controlled drug delivery into the cochlea. The pump integrates drug storage and electronic dosing control, providing precise and sustained drug release for inner ear treatments.	[125]
Hearing loss	Dexamethasone	Melt extruded polymeric implant	Intracochlear	This research investigates PLGA-based implants for intracochlear delivery of dexamethasone. It discusses the challenges of ensuring effective drug release and provides solutions for improving implant performance in treating hearing loss.	[126]
Inner ear disorders	Coumarin-6	Nanoparticles	Intracochlear	This study focuses on the use of PLGA nanoparticles and cell-penetrating peptides for targeted intracochlear drug delivery. By optimizing the nanoparticle properties, the research aims to enhance drug penetration and therapeutic effectiveness for inner ear disorders.	[127]
Hearing loss	Dexamethasone	Hydrogel	Intracochlear	This article evaluates the potential of a hydrogel reservoir for continuous intracochlear delivery of dexamethasone to treat hearing loss. The hydrogel system provides sustained drug release, ensuring prolonged therapeutic effects in the inner ear.	[128]
Hearing loss	Fluticasone propionate	Polymer-coated fluticasone propionate crystals	Intracochlear	This study explores the use of polymer-coated fluticasone propionate crystals for prolonged intracochlear drug delivery. The system provides stable, long-term drug release, offering potential for sustained treatment of hearing loss.	[129]
Hearing loss	Dexamethasone	Microneedle	Intracochlear	This research evaluates the physiological effects of intracochlear dexamethasone-loaded microneedles in guinea pigs. The study provides insights into the safety and effectiveness of dexamethasone microneedles.	[130]

**Table 5:** Recent patents on otic drug delivery systems.

**Patent Application Publication**	**Disease**	**Drug**	**Patent Details**	**References**
KR20220169723A	Hearing loss	Dexamethasone	This patent covers a dexamethasone nanosuspension for drug delivery, designed to effectively treat hearing loss through targeted and sustained release of the drug.	[95]
US20150297588A1	Otomycosis and otitis externa	Antibacterial, antifungal, anti-inflammatory agent	This patent covers the development of sterile otic formulations for treating otomycosis and otitis externa, using a combination of antibacterial, antifungal, and anti-inflammatory agents. These formulations aim to provide effective treatment for ear infections while maintaining sterility.	[131]
EP2802209A1	Otomycosis and otitis externa	Fluoroquinolone, Corticosteroid and antifungal (polyene, triazole, thiocarbamate)	This patent details otic formulations and associated methods for treating otomycosis and otitis externa. It involves the use of fluoroquinolones, corticosteroids, and antifungal agents (polyene, triazole, thiocarbamate), targeting a range of ear infections.	[132]
US20240285651A1	Otomycosis and otitis externa	Marbofloxacin, terbinafine, and/or clotrimazole and dexamethasone	This invention pertains to otic formulations containing marbofloxacin, terbinafine, clotrimazole, and/or dexamethasone for the treatment of otomycosis and otitis externa, offering a multi-drug approach to ear infections.	[133]
US20140107423A1	Acute otitis media	Antimicrobial agent	This patent describes a system and methods for treating acute otitis media using an antimicrobial agent, focusing on targeted delivery and effective treatment of the condition.	[134]
US10821185B2	Otic diseases and conditions	Triglycerides	This patent introduces triglyceride-based otic formulations for treating various otic diseases and conditions, offering a potential new vehicle for drug delivery to the ear.	[135]
US20230190642A1	Otic diseases and disorders	Growth factors	This formulation incorporates growth factors for treating various otic diseases and disorders, aiming to promote healing and regeneration within the ear.	[136]
US20230122991A1	Otic diseases and disorders	Growth factors	This patent focuses on growth factor-based formulations for treating conditions linked to specific otic events, offering a regenerative treatment approach.	[137]
US9486405B2	Pediatric otic diseases	Antimicrobial agent	This patent outlines methods for treating pediatric otic diseases using antimicrobial agents, focusing on safe and effective treatment for children’s ear conditions.	[138]
US20210393511A1	Otic disorders	Antimicrobial agent	This invention covers otic gel formulations containing antimicrobial agents for treating otitis externa, providing a convenient and effective therapeutic approach.	[139]
US11969501B2	Otic disorders	Immunomodulating agent	This patent presents auris formulations containing immunomodulating agents for treating various otic diseases and conditions, focusing on immune system modulation to aid in treatment.	[140]
US20160038594A1	Otic diseases	Antimicrobial agent	This patent focuses on controlled-release antimicrobial compositions for treating otic disorders, providing long-lasting treatment and targeted drug delivery.	[141]
US20220040096A1	Otic disorders	Immunomodulating agent	This patent focuses on Auris formulations containing immunomodulating agents designed to treat various otic disorders. The formulations aim to regulate immune responses within the ear, potentially improving the treatment of ear infections and inflammation.	[142]
EP 3 795 144 A1	Otic disorders	Paraffinum and Oreganum Oil	This patent involves an ear spray device that dispenses a predefined quantity of an ear protection composition capable of preventing ear canal disorders.	[143]
US8648119B2	Otic disorders	Immunomodulating agent	This patent details controlled-release immunomodulator compositions designed to treat otic disorders by regulating the immune system’s response to infection or inflammation.	[144]
JP2014058557A	Otic disorders	Diazepam or Retigabine	This patent involves formulations containing diazepam or retigabine for treating ear diseases and disorders, potentially addressing conditions like ear-related seizures or neurological issues.	[145]
US20210186943A1	Hearing loss	JNK inhibitor, a TRPV modulator, a Met channel inhibitor, and an otoprotectant	This invention focuses on otic formulations for preventing or treating drug-induced ototoxicity, using a combination of JNK inhibitors, TRPV modulators, Met channel inhibitors, and otoprotectants.	[146]
US20220296637A1	Hearing loss	Sodium thiosulfate	This patent involves the use of a sodium thiosulfate gel as a treatment for preventing or reducing hearing loss, offering a potential solution for ototoxicity or other hearing impairments.	[147]
WO2019154895A1	Hearing loss	PPAR agonist agent	This patent presents a gel formulation containing PPAR agonist agents for preventing or treating hearing loss, focusing on neuroprotective effects to preserve hearing.	[148]
US11467386B2	Hearing loss	Prophylactic agent, a diagnostic agent	This invention details systems and methods for treating hearing loss with prophylactic agents and diagnostic agents, focusing on prevention and early detection of hearing impairment.	[149]

**Table 6:** Recent clinical trial studies on otic drug delivery systems.

NCT Number*	**Local Administration**	**Disease**	**Drug**	**Aim**	**Enrollment**	**Phase**	**Last Known Status**	**Outcomes**
NCT00802529	Transtympanic	Ménière's Disease	Methylprednisolone and Gentamicin	This study compares transtympanic steroids, specifically methylprednisolone and gentamicin, with the standard treatment (transtympanic gentamicin) in refractory unilateral Ménière’s disease.	60	Phase 2 Phase 3	Completed	The study compared transtympanic gentamicin and methylprednisolone for treating refractory Ménière's disease. Both treatments significantly reduced vertigo attacks over 18-24 months, with gentamicin showing a slightly higher reduction (from 19.93 to 2.5 attacks) compared to steroids (from 16.4 to 1.6 attacks). Adverse events were rare, with no serious complications; however, minor issues like otitis media and retinoschisis were reported in a few cases.
NCT06521190	Transtympanic	Cisplatin-induced Hearing Loss	ACOU085	This Phase 2 clinical trial evaluates ACOU085 as a potential drug to prevent hearing loss in testicular cancer patients undergoing cisplatin chemotherapy.	40	Phase 2	Recruiting	Not yet provided.
NCT04129697	Intratympanic	Sudden Hearing Loss	Dexamethasone or methylprednisolone	This study investigates intratympanic steroid administration, including dexamethasone or methylprednisolone, as a rescue treatment for sudden hearing loss.	30	Not applicable	Recruiting	Not yet provided.
NCT04120116	Intratympanic	Sensorineural Hearing Loss	FX-322	This Phase 2 study tests the efficacy of repeat doses of FX-322, a novel drug for sensorineural hearing loss, compared to a placebo.	95	Phase 2	Completed	Results showed no significant improvement in word recognition, words-in-noise performance, or pure-tone audiometry across treatment groups. While some adverse events were reported, no serious safety concerns were identified.
NCT06437054	Intratympanic	Hearing Loss	Dexamethasone, Hyaluronic acid and Indocyanine Green (ICG)	A Phase 1/2 clinical trial to evaluate a mixed injectable delivery vehicle containing dexamethasone, hyaluronic acid, and Indocyanine Green (ICG) for treating intractable hearing loss.	26	Phase 1 Phase 2	Not yet recruiting	Not yet provided.
NCT04794842	Intratympanic	Ménière’s Disease	Tetracaine 5% drops, Phenol 90% concentration	In this early Phase 1 study, the effectiveness of tetracaine 5% drops *versus* focal phenol application for local anesthesia during intratympanic steroid injections is being evaluated.	80	Early Phase 1	Active, not recruıting	Not yet provided.
NCT04798391	Intratympanic	Tinnitus	Dexamethasone	This Phase 4 study investigates the effectiveness of intratympanic dexamethasone combined with lignocaine in managing idiopathic tinnitus.	264	Phase 4	Recruiting	Not yet provided.
NCT00872209	Topical	Otitis Externa	0.3% Ciprofloxacin Ear Drops, 0.3% Ciprofloxacin Foam Otic Cipro	The safety and efficacy of a novel 0.3% ciprofloxacin foam (Otic Cipro) for treating acute diffuse otitis externa of bacterial origin is being evaluated in Phase 1 and 2 trials.	100	Phase 1 Phase 2	Completed	Not provided.
NCT00750633	Topical	Acute Otitis Externa	Moxifloxacin, Dexamethasone phosphate	This Phase 3 study assesses the safety and efficacy of moxifloxacin combined with dexamethasone phosphate in treating acute otitis externa.	990	Phase 3	Completed	The trial has been completed with positive outcomes.
NCT03675841	Topical	Chronic Suppurative Otitis Media	Pazufloxacin Mesilate ear drops	Pazufloxacin Mesilate ear drops are being studied in Phase 1 for safety and tolerability in patients with chronic suppurative otitis media.	33	Phase 1	Recruiting	Not yet provided.
NCT00579189	Topical	Acute Otitis Media	Moxidex, Moxifloxacin	A Phase 3 trial is investigating the safety and effectiveness of Moxidex and moxifloxacin otic solutions in treating middle ear infections in patients with ear tubes.	776	Phase 3	Completed	The study compared Moxidex otic solution (moxifloxacin 0.5% + dexamethasone 0.1%) to moxifloxacin 0.5% solution for treating acute otitis media with otorrhea in children with tympanostomy tubes. Moxidex was more effective, with a median time to otorrhea cessation of 3 days *versus* 5 days for moxifloxacin alone (*p* = 0.001). Clinical cure and microbiological success rates were also higher for Moxidex (84.6% *vs.* 71.5%, *p* = 0.0001), with fewer treatment failures (6.7% *vs.* 14.8%, *p* = 0.0015). Adverse events were similar between groups, with no serious safety concerns.
NCT00579189	Topical	Acute Otitis Media	Moxidex otic solution, Moxifloxacin otic solution	This Phase 2 trial evaluates the safety and efficacy of Moxidex and moxifloxacin otic solutions in treating perioperative tube otorrhea.	303	Phase 2	Completed	Not provided.
NCT04768829	Topical	Otomycosis	*Moringa oleifera* leaf 10 mg/100 ml	This early Phase 1 study investigates the antifungal action of Moringa oleifera leaf extract (10 mg/100 ml) for treating otomycosis.	10	Early Phase 1	Completed	Not provided.

**Note**: *NCT Numbers from ClinicalTrials.gov, National Library of Medicine, 2024.

## LIST OF ABBREVIATIONS

BPB: Blood-perilymph Barrier
OW: Oval Windows
RWM: Round Window Membrane

## STANDARDS OF REPORTING

PRISMA guidelines were followed.
